# Effects of metabolic syndrome on bone mineral density, histomorphometry and remodelling markers in male rats

**DOI:** 10.1371/journal.pone.0192416

**Published:** 2018-02-08

**Authors:** Sok Kuan Wong, Kok-Yong Chin, Farihah Hj Suhaimi, Fairus Ahmad, Soelaiman Ima-Nirwana

**Affiliations:** 1 Department of Pharmacology, Faculty of Medicine, Universiti Kebangsaan Malaysia, Jalan Yaakob Latif, Bandar Tun Razak, Cheras, Kuala Lumpur, Malaysia; 2 Department of Anatomy, Faculty of Medicine, Universiti Kebangsaan Malaysia, Jalan Yaakob Latif, Bandar Tun Razak, Cheras, Kuala Lumpur, Malaysia; Max Delbruck Centrum fur Molekulare Medizin Berlin Buch, GERMANY

## Abstract

This study aimed to evaluate the effects of metabolic syndrome (MetS) induced by high-carbohydrate high-fat (HCHF) diet on bone mineral density (BMD), histomorphometry and remodelling markers in male rats. Twelve male Wistar rats aged 12 weeks old were randomized into two groups. The normal group was given standard rat chow while the HCHF group was given HCHF diet to induce MetS. Abdominal circumference, blood glucose, blood pressure, and lipid profile were measured for the confirmation of MetS. Bone mineral density, histomorphometry and remodelling markers were evaluated for the confirmation of bone loss. The HCHF diet caused central obesity, hyperglycaemia, hypertension, and dyslipidaemia in male rats. No significant difference was observed in whole body bone mineral content and BMD between the normal and HCHF rats (p>0.05). For bone histomorphometric parameters, HCHF diet-fed animals had significantly lower osteoblast surface, osteoid surface, osteoid volume, and significantly higher eroded surface; resulting in a reduction in trabecular bone volume (p<0.05). Feeding on HCHF diet caused a significantly higher CTX-1 level (p<0.05), but did not cause any significant change in osteocalcin level compared to normal rats (p>0.05). In conclusion, HCHF diet-induced MetS causes imbalance in bone remodelling, leading to the deterioration of trabecular bone structure.

## Introduction

Metabolic syndrome (MetS) is a medical condition characterized by the co-existence of at least three of the following characteristics: central obesity, hyperglycaemia, hypertension, high triglyceride, and low high-density lipoprotein (HDL) cholesterol [1]. Being a multifactorial disease, MetS increases the risk of other diseases, such as cardiovascular disease [2], non-alcoholic fatty liver disease [3], type 2 diabetes [4], and cancer [5]. On the other hand, osteoporosis is a common skeletal disease characterized by low bone mass and deterioration of bone microarchitecture, which increases the risk of fragility fracture [6]. It occurs due to an imbalance in skeletal remodelling process, leading to increased bone resorption and decreased bone formation [7]. Osteoporotic fracture has massive health and economic burdens on the patients and to the healthcare system [8]. Recently, there is a debate concerning whether MetS is associated with osteoporosis. Many studies involving animals and humans have been performed to investigate the relationship between MetS and osteoporosis but the findings were heterogeneous [9–14]. Human observational studies revealed that individual with MetS had either higher [9, 12] or lower bone mineral density (BMD) [10, 15–17]. Animal studies also yielded inconsistent results, whereby MetS decreased [18] or had no effect [19] on BMD in animals.

Bone marrow mesenchymal cells have an equal tendency for adipocytes or osteoblasts differentiation. Stem cells initially proliferate and differentiate into pre-osteoblast, then to mature osteoblast to produce bone matrix [20]. Metabolic syndrome favours adipocytes differentiation and suppresses osteoblasts differentiation, leading to lower bone formation activity [21]. This is evidenced by a lower osteocalcin level in rats fed with high-fat/high-sucrose diet for 27 weeks [22]. Chronic inflammation induced by MetS also increases the formation of osteoclasts and its bone resorptive activities. A previous study by Pirih et al showed high-fat diet elevated circulating carboxyl-terminal telopeptides of type 1 collagen (CTX-1) level, a marker of bone resorption, in hyperlipidaemic mice [23]. The changes in bone cell ratios, surface erosion, mineralization and ultimately microarchitecture due to MetS can be studied using bone histomorphometry methods. Static histomorphometry evaluates the quantity of cells (osteoblast and osteoclast), unmineralized bone (osteoid), and extent of bone resorption (eroded surface). Dynamic histomorphometry studies the changes in cellular activity over time. Structural parameters measure the bone volume and structure, which are the end results of bone remodelling process [24]. However, to the best of our knowledge, the effects of MetS on bone histomorphometric indices are not well-established.

The objective of the current study was to explore the effects of MetS on bone health, in terms of bone mineral content (BMC), BMD, bone histomorphometric indices, and bone remodelling markers, using a MetS rat model. We administered a previously validated high-carbohydrate high-fat (HCHF) diet to the rats to induce MetS [25]. This model is more similar with human dietary pattern causing MetS [26]. We hope that the findings of this study can provide a clear picture on the effects of MetS on changes in the ratio of bone cells and their activities, and the resultant skeletal structural alterations. We hypothesized that MetS skewed bone remodelling process towards resorption by increasing osteoclast number and decreasing osteoblast number, which ultimately resulted in bone loss.

## Materials and methods

### Animal experimental design

The protocol of animal experimentation were reviewed and approved by the Universiti Kebangsaan Malaysia Animal Ethics Committee (UKMAEC) (Code: PP/FAR/2015/IMA/20-MAY/679-JUNE-2015-MAY-2017). Twelve male Wistar rats aged 12 weeks old were purchased from Laboratory Animal Resource Unit of Universiti Kebangsaan Malaysia. The rats were kept individually in plastic cages at the vivarium of the Department of Anatomy, Universiti Kebangsaan Malaysia (Kuala Lumpur, Malaysia) with a constant ambient temperature of 25 ± 2°C and an alternated 12-hour light/dark cycle. Upon acclimatization for one week, they were randomly divided into two groups (n = 6/group), namely the normal and the HCHF groups. The normal group were fed with standard rat chow (Gold coin, Malaysia) and tap water. The HCHF group were fed with HCHF diet to induce MetS. The HCHF diet (1 kg) contained 375 g of sweetened condensed milk, 200 g of ghee, 175 g of fructose, 155 g of powdered rat food, 25 g of Hubble Mendel and Wakeman salt mixture and 50 mL of water. The drinking water was supplemented with 25% fructose. All the rats were given *ad libitum* excess to water and their assigned diet. The composition of each diet is listed in Table 1.

**Table 1:** The composition of standard diet and HCHF diet used in this study.

Standard diet		HCHF diet	
Ingredients	Percentage/Amount	Ingredients	Percentage/Amount
Carbohydrate	-	Carbohydrate	46.47%
Fat	3%	Fat	26.95%
		Saturated fat	16%
		Unsaturated fat	10.49%
Protein	21%	Protein	7.26%
Moisture	13%	Moisture	7.07%
Salt mixture	1.2%	Salt mixture	2.69%
Fibre	5%	Fibre	0.78%
Ash	8%	Ash	1.23%
Nitrogen free extract	48.8%	Nitrogen free extract	7.55%

### Metabolic syndrome parameters

All MetS parameters were assessed at week 0, 8 and 16. Calorie intake of animals was calculated as 13.80 kJ/g and 17.81 kJ/g of food for standard rat chow and HCHF diet, respectively. The 25% fructose-supplemented drinking water contained 3.85 kJ/mL [25]. Body weight of animals was recorded. Abdominal circumference was measured using a measuring tape under light sedation. Fasting blood glucose determination was performed after 12 hours of food deprivation. After that, oral glucose tolerance test (OGTT) was performed and the rats were orally administrated with a glucose load (2 g/kg) in 40% glucose solution. Blood samples were taken from tail vein at 0, 30, 60, 90, and 120 minutes. Blood glucose concentrations were measured using ACCU Check Performa glucometer (Roche Diagnostic, USA) and readings were converted into area under the curve (AUC_glucose_) using trapezoidal rule. Systolic and diastolic blood pressure of the rats were monitored using non-invasive tail-cuff system (CODA™, Kent Scientific Corporation, Torrington, CT, USA) after warming the rats for 10 minutes. For the evaluation of lipid profile, blood was collected from the rats, immediately processed into serum (centrifuged at 3,000 rpm for 10 minutes), and stored at -70°C until analysis. Serum triglyceride, total cholesterol, HDL cholesterol, and low density lipoprotein (LDL) cholesterol levels were measured using commercial colorimetric assay kits (BioAssay System, USA).

### Body composition and bone densitometry

**Fig 1:**
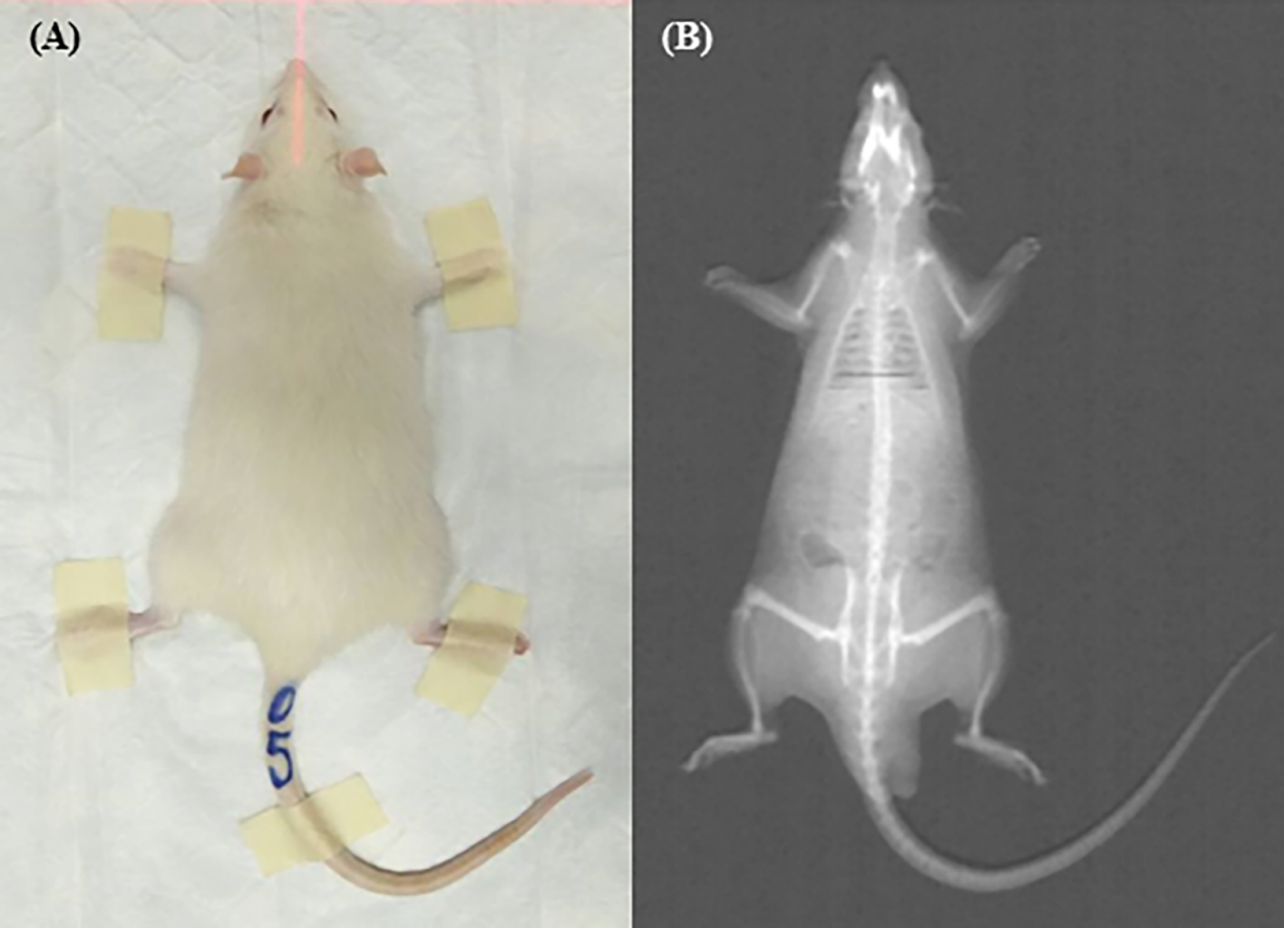
Dual-energy X-ray absorptiometry (DXA) scan. (A) Positioning of a rat during a whole body scan by DXA. Rat was positioned in ventral recumbency on the scan table. (B) Image for total body scan of a rat from DXA scan.

Bone densitometry was conducted under general anaesthesia. The anaesthetized rat was positioned in ventral recumbency on the scan table. All scans were performed using dual-energy X-ray absorptiometry (DXA) to evaluate body composition (fat mass, lean mass, percentage of fat, global BMC, and BMD) of the rats at week 0, 8, and 16 (Fig 1). All DXA scans were analysed using manufacturer’s recommended software (Small Animal Analysis Software, Hologic QDR-1000 System).

### Biochemical assay for bone markers

Osteocalcin (bone formation marker) and CTX-1 (bone resorption marker) were measured using commercial enzyme-linked immunosorbent assay (ELISA) kit as per manufacturer’s instructions (Immunodiagnostic Systems, Tyne and Wear, UK).

### Bone processing and histomorphometric analysis

At the end of the study, rats were euthanized with overdoses of ketamine-xylazine combinations (Dose: ketamine, 300 mg/kg; xylazine, 30 mg/kg) and femur was harvested for bone histomorphometry analysis. Left femurs were sawed into halves. For undecalcified section, bone samples were fixed in 70% alcohol. For the decalcified section, bone samples were fixed in 10% neutral buffered formalin and then decalcified with 10% ethylenediaminetetraacetic acid (EDTA) solution for two months.

For structural bone histomorphometry measurements, the undecalcified bone samples was embedded in polymethyl methacrylate (Polysciences, USA) and cut longitudinally into 5 μm-thick sections using microtome (Leica RM2235, Nussloch, Germany). The sections were stained using von Kossa method and analysed using an image analyser (Nikon Eclipse 80i, Tokyo, Japan) with automated image analysis software (MediaCybernetics Image Pro-Plus, Rockville, MD, USA). The structural parameters included bone volume / tissue volume (BV/TV, unit = %), trabecular bone thickness (Tb.Th, unit = μm), trabecular bone number (Tb.N, unit = μm^-1^), and trabecular bone separation (Tb.Sp, unit = μm) were measured under 100X magnification.

For static bone histomorphometry measurements, the decalcified bone samples were embedded in paraffin wax, sectioned longitudinally into a thickness of 5 μm using microtome (Leica RM2235, Nussloch, Germany), and stained using haematoxylin and eosin. The sections were visualized under a light microscope (Nikon Eclipse 80i, Tokyo, Japan). The static parameters included osteoblast surface (Ob.S/BS, unit = %), osteoclast surface (Oc.S/BS, unit = %), eroded surface (ES/BS, unit = %), osteoid surface (OS/BS, unit = %), and osteoid volume (OV/BV, unit = %) were measured under 200X magnification. For dynamic bone histomorphometry measurements, calcein (20 mg/kg body weight) (Sigma-Aldrich, St Louis, MO, USA) were injected intraperitoneally for fluorochrome labelling of the bone on days 9 and 2 before the rats were sacrificed. The unstained sections (thickness: 5 μm) of undecalcified bone samples were photographed using a fluorescent microscope (Nikon Eclipse 80i, Tokyo, Japan) equipped with an automated image analysis software (MediaCybernetics Image Pro-Plus, Rockville, MD, USA). The dynamic parameters included single-labelled surface (sLS/BS, unit = %), doubled-labelled surface (dLS/BS, unit = %), mineralizing surface (MS/BS, unit = %), mineral apposition rate (MAR, unit = μm/day), and bone formation rate (BFR, unit = μm^3^/μm^2^/day) were assessed under 200X magnification. The region of interest for all histomorphometric parameters was the secondary spongiosa in the metaphyseal cancellous region (1–3 mm distal to the growth plate).

### Statistical analysis

The statistical analysis was performed using Statistical Package for Social Sciences (SPSS) version 20 software (IBM, Armonk, NY, USA). Relative fold change of MetS parameters and body composition were calculated as the ratio of the final reading over the initial reading. General linear (repeated) measure was used to analyse the significant differences for MetS parameters, body composition, and bone markers between the study groups. One-way analysis of variance (ANOVA) was used to assess the significant difference for all the bone histomorphometric parameters between the normal and HCHF groups. All data are presented as mean ± standard error of the mean (SEM). The statistical differences were considered significant at p<0.05.

## Results

**Fig 2:**
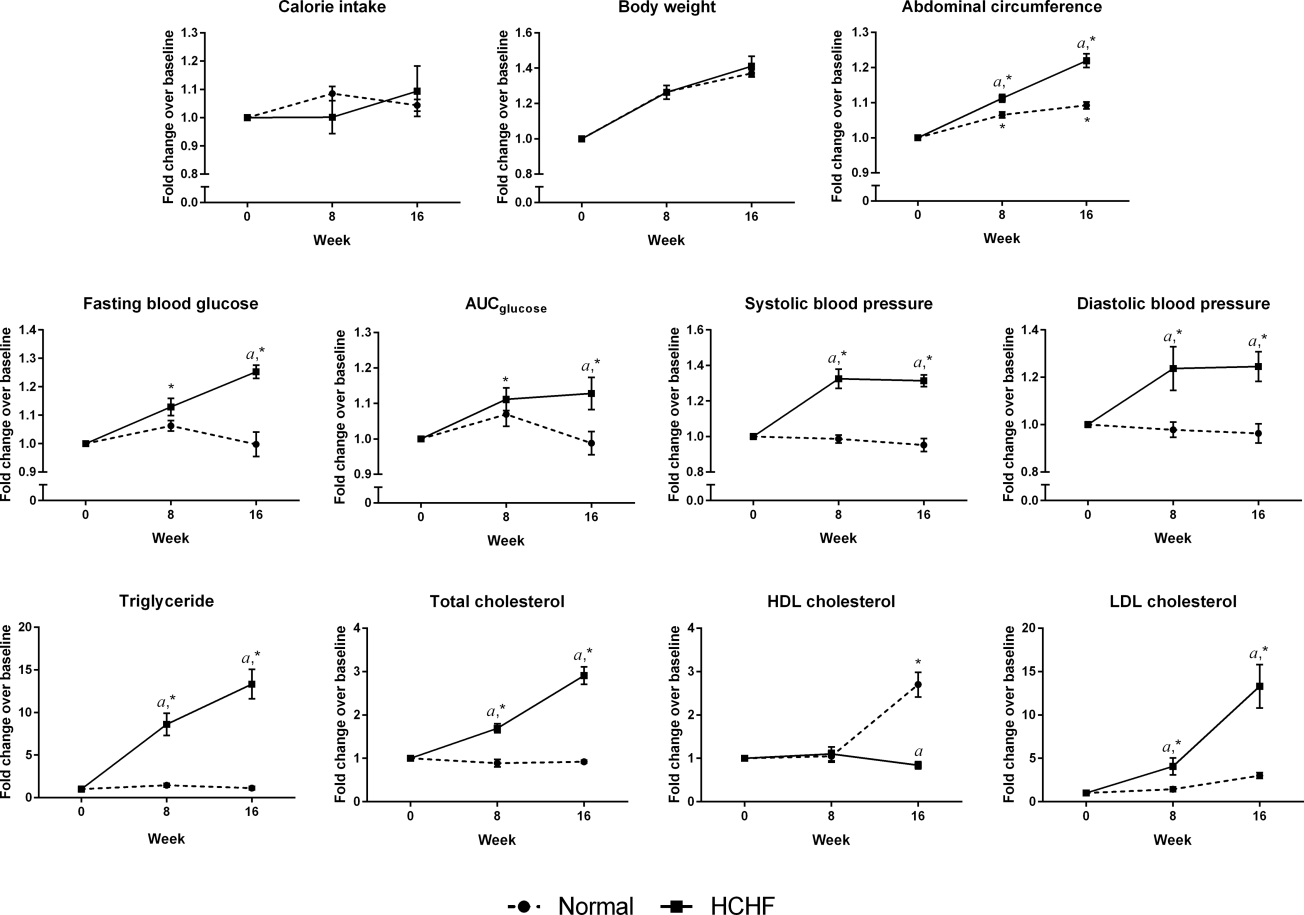
Evaluation of MetS parameters in the two experimental groups. Relative fold change of MetS parameters (calorie intake, body weight, abdominal circumference, fasting blood glucose, AUC_glucose_, systolic blood pressure, diastolic blood pressure, triglyceride, total cholesterol, HDL cholesterol, and LDL cholesterol) over baseline at week 0, 8, and 16. The data were expressed as mean ± SEM. Letter ‘*a*’ indicated significant difference (p<0.05) compared to the normal group and ‘*’ indicated significant difference (p < 0.05) compared to week 0.

**Fig 3:**
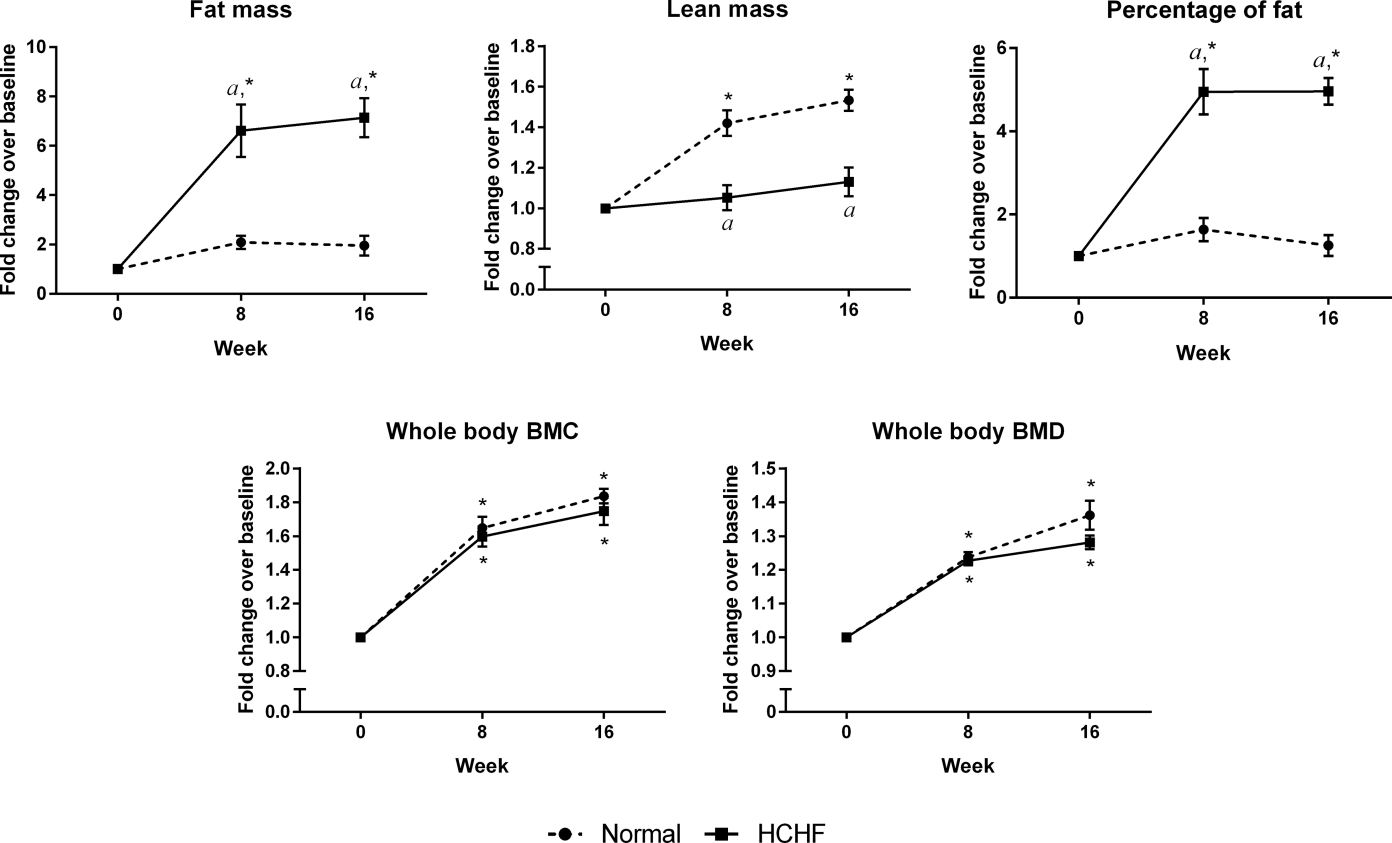
Evaluation of body composition by DXA in the two experimental groups. Fat mass, lean mass, percentage of fat, whole body BMC, and BMD in the normal and HCHF rats at week 0, 8, and 16. The data were expressed as mean ± SEM.

The calorie intake and body weight were similar between the normal and HCHF animals (p>0.05). The HCHF rats had significantly higher abdominal circumference, fasting blood glucose, blood pressure, glucose intolerance, triglyceride, total cholesterol, and LDL cholesterol but significantly lower HDL cholesterol level compared to the normal rats at week 16 (p<0.05) (Fig 2). Results of body composition obtained from DXA scan revealed that rats fed with HCHF diet had higher fat mass and percentage of fat, but lower lean mass as compared to the normal rats (p<0.05) (Fig 3). These findings indicated the occurrence of MetS in animals after feeding with HCHF diet.

**Fig 4:**
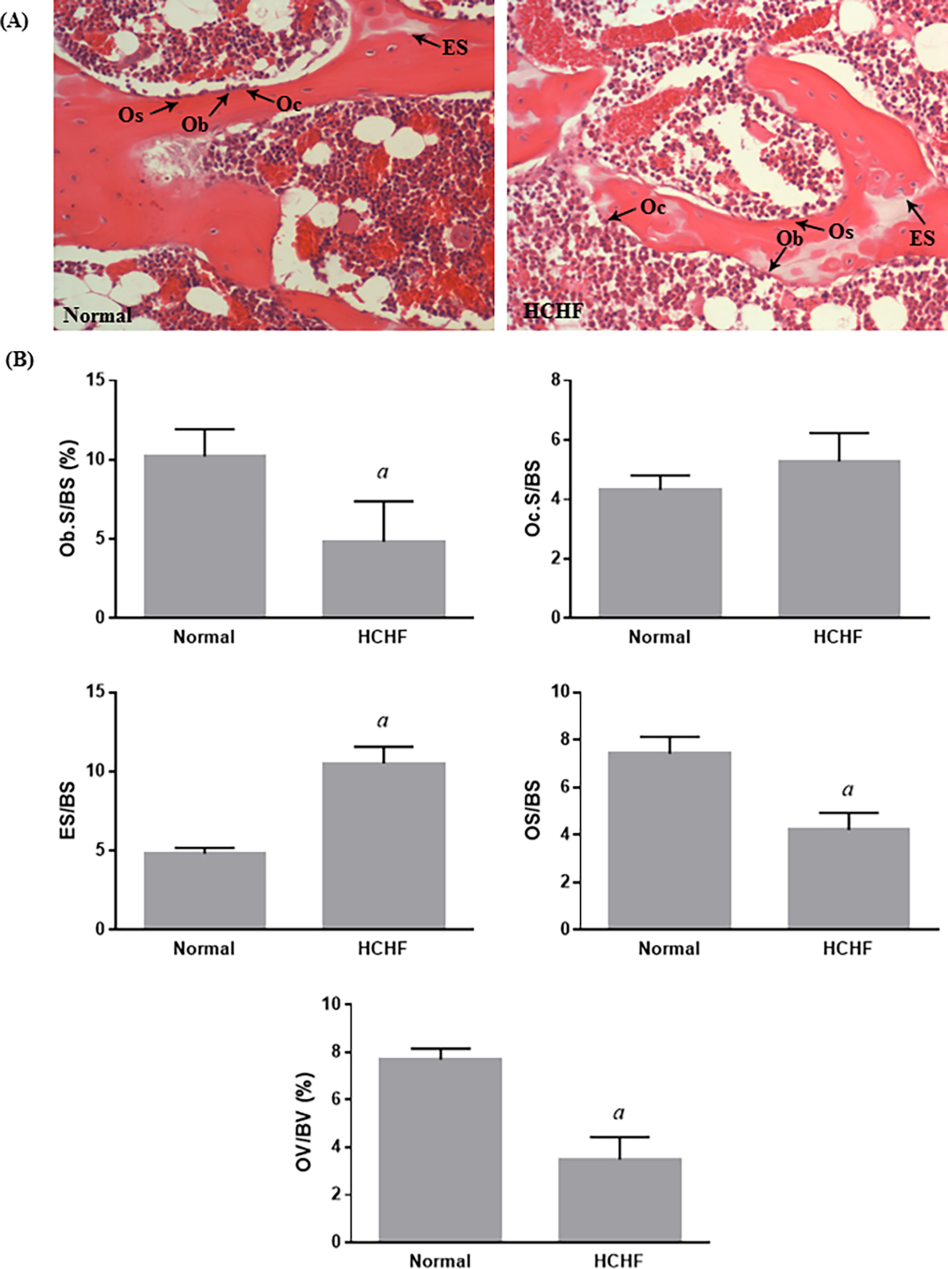
Evaluation of static bone histomorphometric parameters in the two experimental groups. (A) Representative micrographic photos of decalcified trabecular bone in the normal and HCHF rats stained with haematoxylin and eosin (200X magnification). (B) The static indices included Ob.S/BS, Oc.S/BS, ES/BS, OS/BS, and OV/BV. The data were expressed as mean ± SEM. Letter ‘*a*’ indicated significant difference (p<0.05) compared to the normal group. Abbreviations: ES = eroded surface; Ob = osteoblast; Oc = osteoclast; Os = osteoid.

**Fig 5:**
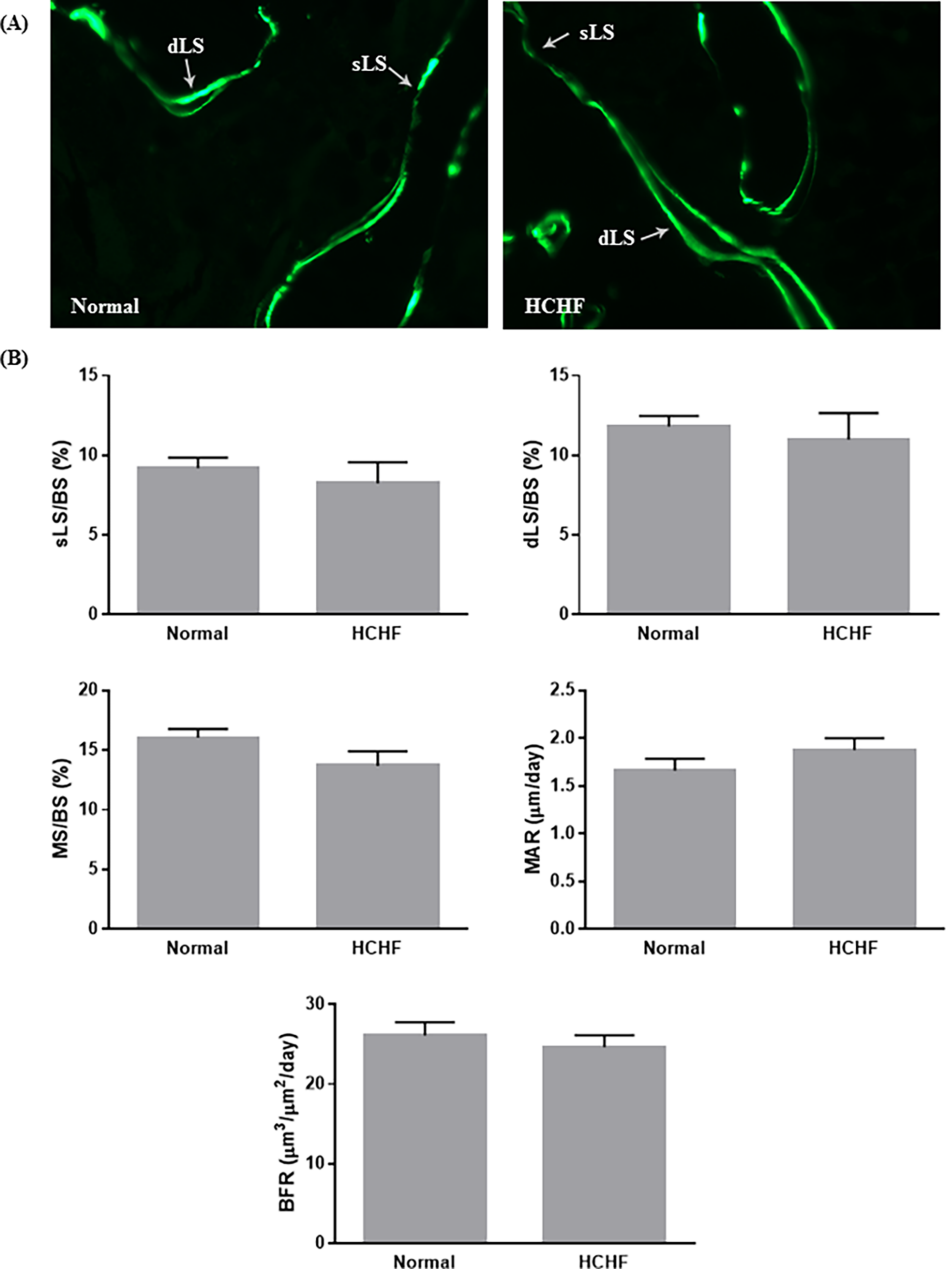
Evaluation of dynamic bone histomorphometric parameters in the two experimental groups. (A) Representative micrographic photos of undecalcified trabecular bone in the normal and HCHF rats without staining (200X magnification). (B) The dynamic indices included sLS/BS, dLS/BS, MS/BS, MAR, and BFR. The data were expressed as mean ± SEM. Abbreviation: dLS = double-labelled surface; sLS = single-labelled surface.

**Fig 6:**
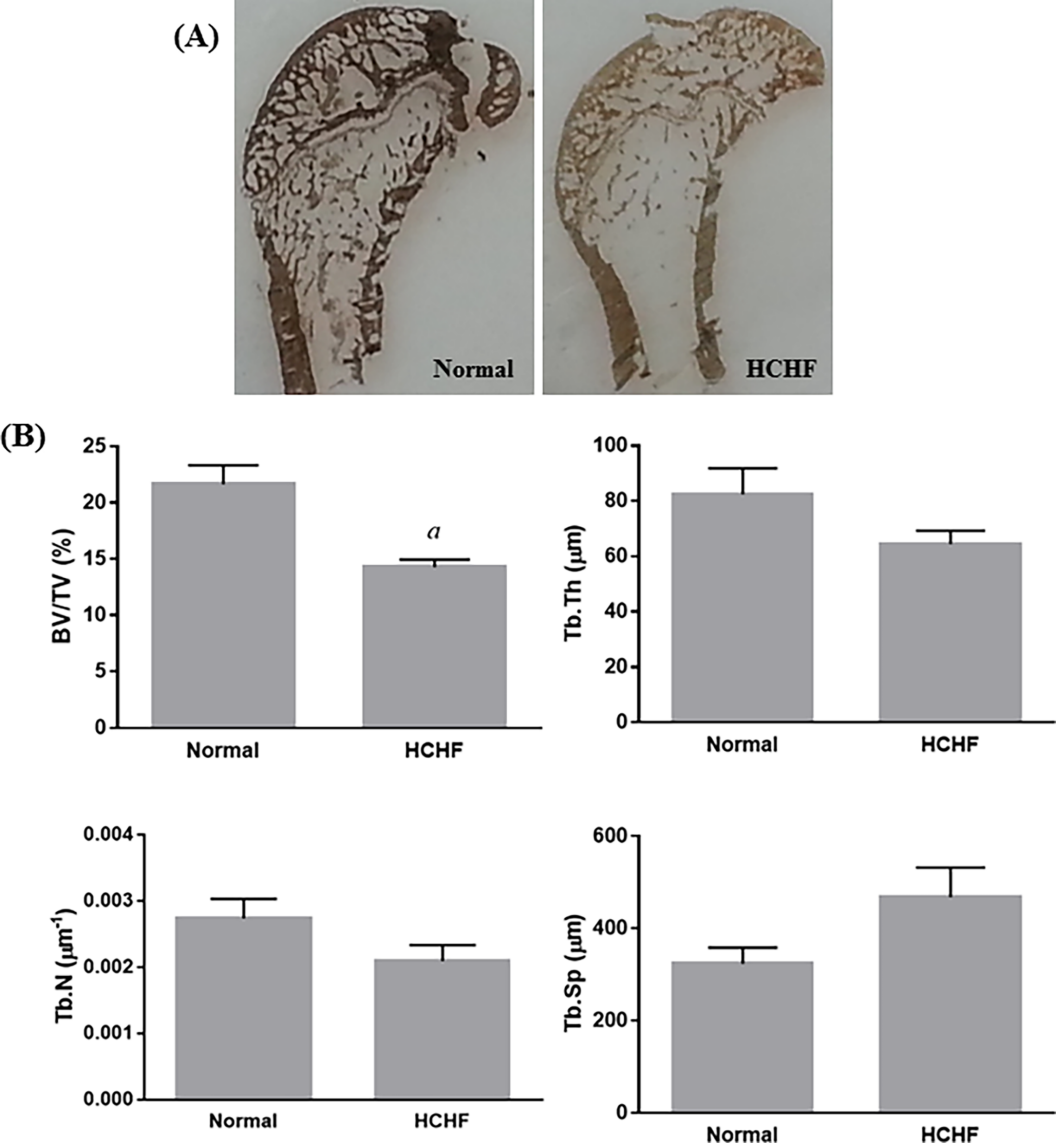
Evaluation of structural bone histomorphometric parameters in the two experimental groups. (A) Sections of distal femur in the normal and HCHF rats stained with von Kossa staining (4X magnification). (B) The structural indices included BV/TV, Tb.Th, Tb.N, and Tb.Sp. The data were expressed as mean ± SEM. Letter ‘*a*’ indicated significant difference (p<0.05) compared to the normal group.

Twenty weeks of HCHF diet did not cause any significant difference to the whole body BMC and BMD in animals (p>0.05) (Fig 3). The static indices indicated that HCHF diet caused significant decreases in ObS/BS, OS/BS, OV/BV, and significant increase in ES/BS (p<0.05) (Fig 4). Meanwhile, no significant differences were observed in all dynamic parameters (sLS/BS, dLS/BS, MS/BS, MAR, and BFR) between the two study groups (Fig 5). For bone structural histomorphometric parameters, HCHF diet-fed animals had significantly reduced BV/TV compared to the standard diet-fed animals (p<0.05), but Tb.Th, Tb.N, and Tb.Sp did not show significant changes (p>0.05) (Fig 6).

**Fig 7:**
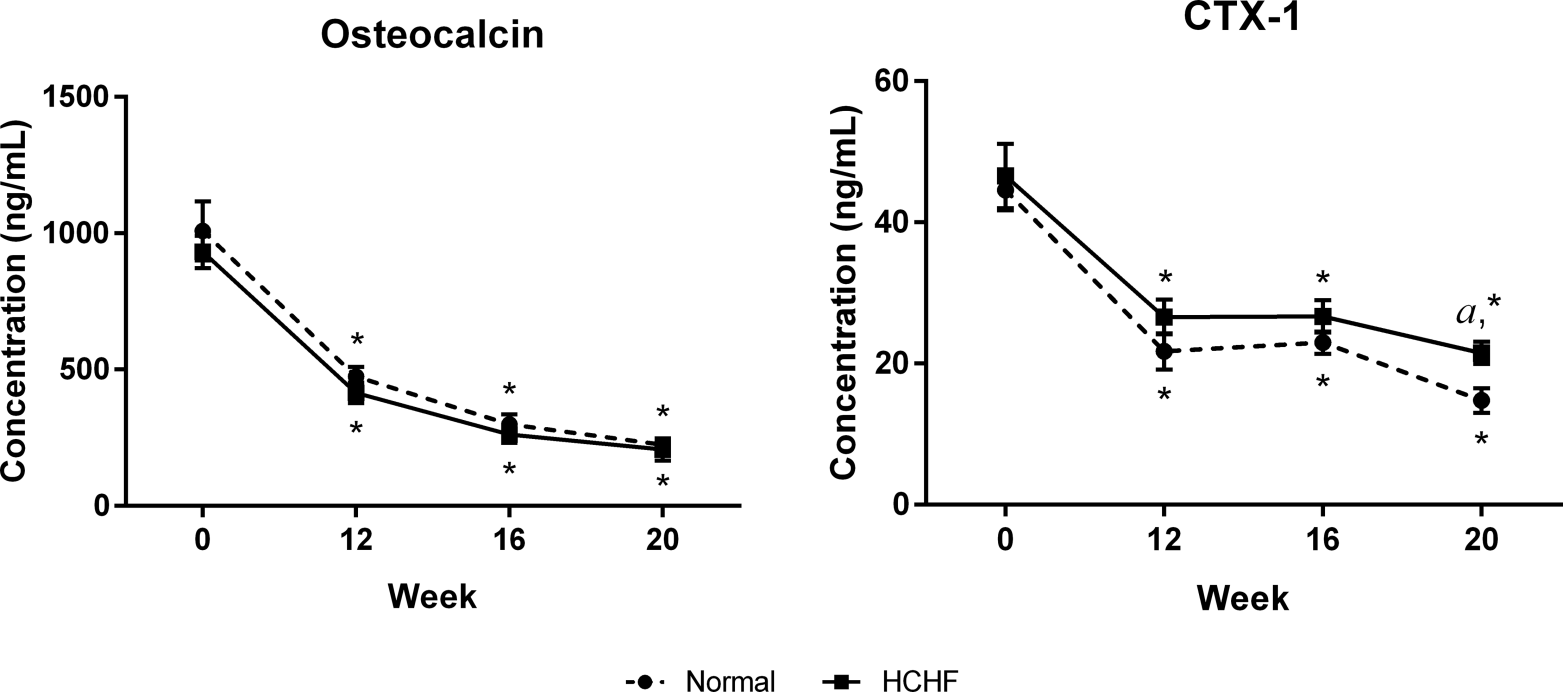
Evaluation of bone remodelling markers in the two experimental groups. Levels of serum osteocalcin and CTX-1 in the normal and HCHF rats at week 0, 12, 16, and 20. The data were expressed as mean ± SEM. Letter ‘*a*’ indicated significant difference (p<0.05) compared to the normal group and ‘*’ indicated significant difference (p < 0.05) compared to week 0.

The circulating osteocalcin level did not show any significant difference throughout the study period (p>0.05). However, the circulating CTX-1 level was significantly increased after 20 weeks of HCHF diet compared to the normal animals (p<0.05) (Fig 7).

## Discussion

The purpose of this study was to investigate the effects of HCHF diet-induced MetS on BMC, BMD, bone histomorphometric parameters, and bone markers in male rats. Metabolic syndrome was successfully induced by HCHF diet, evidenced by the fulfilment of all MetS criteria, including central obesity, hyperglycaemia, hypertension, and dyslipidaemia. Although the calorie intake and body weight were comparable between the normal and HCHF animals, results obtained from DXA scan demonstrated that the HCHF rats had higher fat mass, percentage of fat, and lowered lean mass compared to the normal rats. The possible explanation is that each unit of calorie intake by the HCHF animals contained the high content of fat and low content of protein which contributed to accumulation of fat in animals. Besides, the lean mass is denser than fat mass indicating that lean mass is heavier than fat mass in an equal volume. For bone parameters, we found that HCHF diet had no effects on whole body BMC and BMD. Findings from bone histomorphometric analysis revealed that HCHF diet caused reductions in Ob.S/BS, OS/BS, OV/BV, BV/TV, and an increment in ES/BS. Surprisingly, HCHF diet had no effect on Oc.S/BS, sLS/BS, dLS/BS, MS/BS, MAR, BFR, Tb.Th, Tb.N, and Tb.Sp. Feeding on HCHF diet also caused a significant increase in CTX-1 level but no significant difference in osteocalcin level compared to animals fed with standard diet.

To obtain a preliminary picture of bone health, bone densitometry was used to measure whole body BMC and BMD in the two experimental groups. Our current findings showed no difference in whole body BMC and BMD between the normal and HCHF rats. This observation might be due to the low resolution of DXA method for small animal. It also does not differentiate between cortical and trabecular bone as our earlier study found that HCHF diet caused a deterioration of trabecular bone microstructure, but did not affect the cortical bone structure in male rats [27]. Results from present study were in line with previous study by Doucette et al, whereby BMD of the mice fed with high-fat diet for 12 weeks was similar with the normal control [28]. Another study also found that BMD of animals was not significantly different in both the high-fat diet and control groups [29]. Contrary to these results, study by Malvi et al showed that high-fat diet supplemented with ground nut and coconut increased BMD and BMC in rats. Authors suggested that the lipid components (unsaturated fatty acids) present might enhance the bone-related parameters [30]. Some other *in vivo* studies further demonstrated the differential effects of diet containing saturated and unsaturated fatty acids on BMD [31, 32]. These studies indicated that diet with high content of saturated fatty acids adversely affects bone in animals, but not in diet containing unsaturated fatty acids [31, 32]. Combined with all these findings from previous literature, we postulated that difference in the types of fatty acids in diet might impose different effects on bone.

Our results from bone histomorphometric analysis showed that animals fed with HCHF diet had significantly decreased Ob.S/BS and comparable Oc.S/BS at the trabecular bone compared to the normal control, which resulted in increased ES/BS, as well as decreased OS/BS and OV/BV. These changes in the static parameters potentially led to a reduction in BV/TV. There were trends of reductions in Tb.Th, Tb.N, and increment in Tb.Sp in the animals fed with HCHF diet, but the changes did not reach statistical different. Our findings was further supported by previous comparable studies conducted by Felice and co-workers, demonstrating that fructose-induced MetS caused deleterious effects on bone microarchitecture and impaired bone regeneration [21, 33]. Bone resorption precedes formation in bone remodelling event under normal condition [34]. The deterioration of bone structure in HCHF animals could be due to the imbalance of osteoblast and osteoclast activity, resulting in increased creation of resorption cavities and decreased matrix synthesis. These changes conferred detrimental effects on the cancellous bone structure [35]. Dynamic parameters define bone turnover rate. Surprisingly, there was no significant difference in all dynamic parameters between the two study groups, including sLS/BS, dLS/BS, MS/BS, MAR, and BFR. These findings could be attributed to the increased bone formation activity of the remaining osteoblasts to compensate the increased resorption activity by the osteoclasts. These findings from the dynamic parameters also explain that HCHF diet caused significant decrease only in BV/TV but not in other structural parameters. However, similar study on the effects of high-fat/high-fructose diet on bone histomorphometric analysis is limited.

Serum CTX-1 has been reported to be more specific and sensitive markers of bone resorption compared to other resorption markers [36]. In our study, we found that HCHF diet caused a significant increase in CTX-1 level. Comparable results were obtained from previous animal study that high-fat diet increased bone resorption marker, CTX-1 in hyperlipidaemic mice [23]. Although Oc.S/BS did not increase significantly, the increase in serum CTX-1 indicated an increase in the osteoclastic bone resorption in HCHF rats. Osteocalcin is a non-collegenous protein, secreted by osteoblasts, and has a role in calcium binding and stabilization of hydroxyapatite in the bone matrix [37]. Osteocalcin is a global marker for bone remodelling. Our present findings demonstrated that HCHF rats had significant decrease in Ob.S/BS but serum osteocalcin level did not show any significant changes as compared to normal rats. This observation could be explained by the reduction in osteoblastic differentiation and proliferation. However, the bone formation activity by the osteoblasts increased in response to the increase in bone resorption, indicating the high bone turnover rate in the HCHF animals. Besides, our data on serum osteocalcin level also further supported the dynamic histomorphometric data, whereby osteocalcin level often correspond to BFR assessed by histomorphometry [38]. Our results were in concordance with previous study which indicated that high-fat diet feeding for 11 weeks did not affect serum concentration of osteocalcin in mice [39].

Overall, our study showed that MetS could cause an increase in bone resorption activity by the osteoclasts without changing their number. MetS also decreased the osteoblast number, but the remaining cells compensated the bone resorption process by increasing their activity. The net result of the imbalance in bone remodelling was a decreased bone volume, although the change in bone geometry was minimal. The increase of bone resorption might be attributed to the transient chronic inflammation during MetS. The increase in circulating inflammatory mediators produced by adipocytes promotes osteoclast activities through the activation of receptor activator of NF-κB (RANK) / receptor activator of NF-κB ligand (RANKL) / osteoprotegerin (OPG) pathway [40]. On the other hand, the increasing bone formation may be attributed to the hormonal changes during MetS. The levels of hormone leptin and insulin are highly correlated with MetS, whereby these hormones have been reported to have an important role on bone. Leptin acts through peripheral pathway to increase osteoblast activities [41]. Besides, the inhibition of cortisol and glucocorticoids by leptin promotes bone health [42]. Insulin binds to its receptor on osteoblast and stimulates bone formation via insulin receptor substrate (IRS-1 and IRS-2) [43]. Thus, we speculated that MetS might have dual effects on bone but further evidence is needed to validate this.

This study describes the effects of HCHF diet-induced MetS on BMC, BMD, bone histomorphometric indices, and bone remodelling markers in rats. It attempts to resolve the complex effects of MetS on bone using bone histomorphometry, mainly through the evaluation of the changes in cell numbers, bone erosion and mineralization. We demonstrated that HCHF diet caused imbalance in bone cell activities responsible for the regulation of skeletal remodelling. We also acknowledged the limitations of this study. Firstly, better results might be obtained by evaluating the BMC and BMD at specific sites, particularly at weight-bearing bones such as femurs and tibias. Secondly, the levels of undercarboxylated and carboxylated osteocalcin were not evaluated. In addition, mechanical loading on bone and the evaluation of inflammatory mediators in animals were not performed in this study. Our findings warrant further studies on the investigation of possible underlying mechanism, particularly the inflammatory response, responsible for the deterioration of trabecular bone, which is currently not fully understood.

## Conclusion

MetS induced by HCHF diet compromises bone structure in male rats. This is due to an imbalance between osteoblastic and osteoclastic activities, reflected by the elevation of bone resorption marker. Further studies are needed to validate the underlying mechanisms causing this imbalance.
